# Mitochondrial genome of *Pseudopotamilla reniformis* (Annelida: Sabellidae)

**DOI:** 10.1080/23802359.2022.2164230

**Published:** 2023-01-15

**Authors:** Dmitry Fedorov, Dmitry Knorre, Glafira Kolbasova, Tatiana Neretina

**Affiliations:** a Skolkovo Institute of Science and Technology, Moscow, Russia; b Belozersky Institute of Physico-Chemical Biology, Lomonosov Moscow State University, Moscow, Russia; c Pertsov White Sea Biological Station, Lomonosov Moscow State University, Moscow, Russia; d Institute for Information Transmission Problems (Kharkevich Institute), Russian Academy of Sciences, Moscow, Russia

**Keywords:** Mitochondrial genome, Annelida, polychaete, phylogeny

## Abstract

Here, we report the complete mitochondrial genome of sabellid *Pseudopotamilla reniformis* (Bruguière, 1789) (16,408 bp) and comprised of two ribosomal RNAs, the ubiquitous set of 13 protein-coding sequences, and 22 tRNAs. The order of protein-coding genes is consistent with the proposed conserved pattern, which contradicts recent discovery in other members of the family (*Sabella spallanzanii* in Daffe et al., 2021 and *Bispira melanostigma* in Hornfeck et al., 2022).

Sabellida includes the most specialized tube-dwelling filter-feeding annelids. All the three families within Sabellida, Sabellidae, Serpulidae, and Fabriciidae, form a monophyletic group, well supported by morphological and molecular data (Tilic et al. 2020). *Pseudopotamilla reniformis* is a sabellid, widely spread in boreal and arctic seas, forming dense aggregations of leathery tubes on hard substrates in the upper subtidal zone (Ushakov 1955; Kolbasova et al. 2013). This species has a high regeneration ability and intensively reproduces asexually via architomy (Kolbasova et al. 2013). Being easily maintained in marine aquaria, *P. reniformis* is a convenient species for experimental studies addressing both cellular and molecular mechanisms of regeneration and asexual reproduction in Annelida.

All *P. reniformis* individuals were sampled by scuba diving near the White Sea Biological Station of Moscow State University (66°33.17′N, 33°07′E) in August 2021. The specimen, which mitochondrial genome was sequenced, was deposited at the White Sea Biological Station of Moscow State University (http://wsbs-msu.ru, Glafira Kolbasova, voucher no. ZMMU MSU WS0617V). All research reported here has been conducted in an ethical and responsible manner and is in full compliance with all relevant codes of experimentation and legislation. Ethics approval has been obtained from the Biological Faculty of Moscow State University Bioethics Committee.

Mitochondrial DNA was extracted from pieces of tissue (up to 25 μg) using the Diatom™DNA Prep 100 kit (Isogene Lab, Moscow, Russia) according to the manufacturer’s protocol. The paired-end 250 + 250 library was prepared for Illumina HiSeq2000 according to the manufacturer’s protocol (Illumina, San Diego, CA) (Figure 1).

**Figure 1.**
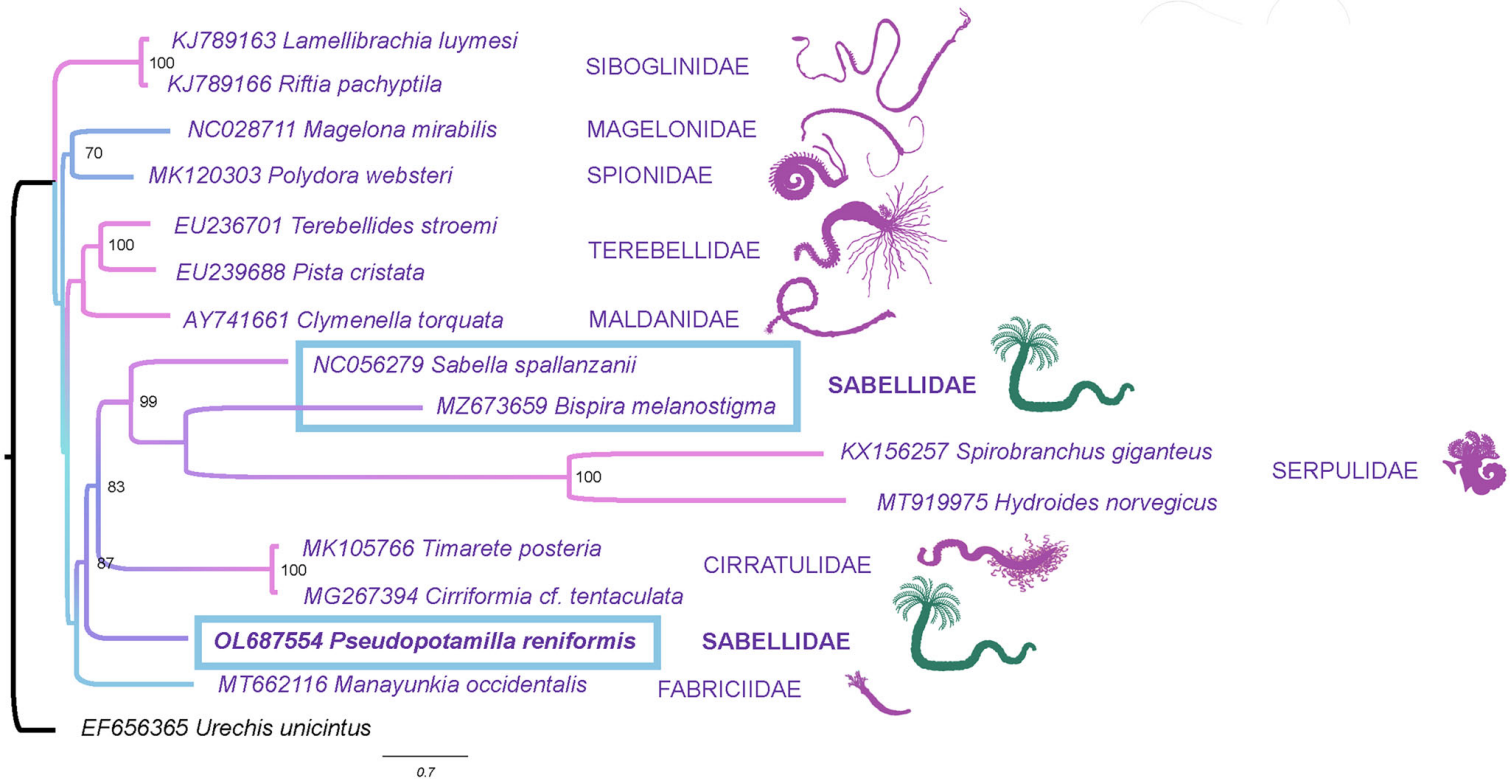
Maximum-likelihood (ML) tree based on the concatenated nucleotide sequences of 12 mitochondrial protein-coding genes. Bootstrap support values are indicated at each node. *Urechis unicinctus* (EF656356) was chosen as an outgroup.

The obtained Illumina genomic paired-end reads quality was measured with FastQC v.0.11.9 and further process of trimming was done in Trimmomatic (v.0.39) (Bolger et al. 2014). After the trimming, the 8,945,967 reads were successfully used in assembly.

The complete circular mitochondrial genome was *de novo* assembled from trimmed Illumina reads with SPAdes (v.3.15.2) (Bankevich et al. 2012) and Novoplasty (v.4.0) (Dierckxsens et al. 2016). The cytochrome oxidase 1 seed gene sequence (HQ024218.1) was chosen to be the seed for the Novoplasty assembly. Both assemblers recovered the identical circular mitochondrial contig. The average coverage for this contig was 162. The mitochondrial chromosome was annotated with the MITOS pipeline (Bernt et al. 2013).

Protein sequences of 11 protein-coding genes (excluding ATP-synthase 8 subunit) of the assembled mitochondrial genome were aligned using ClustalW (Larkin et al. 2007) with protein sequences of 14 other polychaetes, which were downloaded from GenBank. The following alignments then were concatenated into one file, and stable phylogenetic positions were chosen using Gblocks (v.0.91b) (Castresana 2000). The ML tree was generated in the IQtree (v.1.6.12) (Nguyen et al. 2015; Kalyaanamoorthy et al. 2017) with a model (mtZOA + R4) and 1000 bootstrap replicates with the *Urechis unicinctus* (Drasche, 1880) as an outgroup.

The mitochondrial genome of *P. reniformis* is 16,408 bp long. The GC content is 40.5% which is more significant than relative species. AT skew is −0.012.

We identified 13 protein-coding genes, two rRNA genes, and 23 tRNA genes with trnC duplicated. We also detected an AT-rich structured region in proximity to the *ATP8* gene and lysine tRNA, which can be a predictor of mitochondrial origin of replication.

The ML phylogenetic tree built based on 12 amino acid sequences of mitochondrial protein-coding genes (ATP8-synthase gene was excluded because it was not present in all GenBank annotations) shows the results significantly different from those shown by current transcriptomic trees (Tilic, Sayyari, et al. 2020). Although Fabriciidae was a basal branch in position similar to those recovered in other studies (Tilic, Sayyari, et al. 2020), the Sabellidae was not recovered as a monophyletic group according to our data and those of (Hornfeck et al. 2022). However, monophyly of Sabellida is supported with numerous morphological and molecular apomorphies (Tilic, Sayyari, et al. 2020). Moreover, Cirratulidae was nested within the Sabellida, which contradicts all previous morphological and molecular annelid phylogenies (Meyer 1893; Ushakov 1955; Rouse and Fauchald 1997; Bleidorn et al. 2003; Weigert and Bleidorn 2016; Weigert et al. 2016; Tilic, Sayyari, et al. 2020). The gene arrangement pattern in Sabellidae varies between species thus both conserved (Weigert et al. 2016) and divergent gene (Daffe et al. 2021; Hornfeck et al. 2022) order can be observed. The gene order found in *P. reniformis* could be classified as conserved, while modified gene orders were found in both *Sabella spallanzanii* (Daffe et al. 2021) and *Bispira melanostigma* (Hornfeck et al. 2022). Moreover, the order of protein-coding genes varies and is highly divergent in the Serpulidae (Seixas et al. 2017; Sun et al. 2021), which could result in the phylogeny discrepancies. The uncertainty of the phylogenetic position of the Cirratulidae shows a need for additional mitochondrial genomes accompanied by nuclear genomic data.

## Data Availability

The genome sequence data that support the findings of this study are openly available in GenBank of NCBI at https://www.ncbi.nlm.nih.gov/ under the accession no. OL687554. The associated BioProject, SRA, and Bio-Sample numbers are PRJNA810340, SRR18156154, and SAMN26245631, respectively.
